# Solving the mystery of persistent isolated aspartate aminotransferase elevation: Macro-AST as a decisive diagnostic consideration

**DOI:** 10.3389/fcimb.2026.1895517

**Published:** 2026-10-07

**Authors:** Lina Sun, Yao Liu, Xue Yang, Fangyuan Gao, Lei Sun, Yu Lu, Yanqiu Li, Ying Feng, Xianbo Wang

**Affiliations:** 1 Center of Integrative Medicine, Beijing Ditan Hospital, Capital Medical University, Beijing, China; 2 National Center for Infectious Diseases, Beijing Ditan Hospital, Capital Medical University, Beijing, China; 3 Department of Pathology, Beijing Ditan Hospital, Capital Medical University, Beijing, China

**Keywords:** AST recovery rate, immune dysregulation, macro-AST, PEG precipitation, PPA%

## Abstract

**Background:**

Persistent isolated elevation of aspartate aminotransferase (AST) often poses a diagnostic challenge. Macro-AST, though underrecognized, is a benign cause that may avoid unnecessary investigations.

**Aim:**

To characterize the clinical and laboratory features of macro-AST in Chinese adults and to describe our experience with the PEG precipitation method in identifying this condition.

**Methods:**

Clinical data from patients presenting with persistent isolated AST elevation at Beijing Ditan Hospital, Capital Medical University, between February and August 2025 were analyzed. All patients underwent comprehensive laboratory tests (including routine biochemistry, muscle enzymes and autoantibodies), imaging studies (abdominal ultrasound and transient elastography), and liver biopsy. After excluding other definitive diseases, macro-AST was confirmed using the PEG precipitation method. Sera from one healthy control and one disease control (with chronic active hepatitis B) were also collected.

**Results:**

Three patients (2 female, 1 male) were confirmed with macro-AST. Immunological evaluation revealed heterogeneous serological features among the three patients, including positive autoantibodies and variably elevated immunoglobulin levels in a subset of cases. The persistent isolated AST elevation was not adequately explained by any histological finding. Following PEG precipitation, the AST recovery rate was considerably lower in macro-AST patients (mean 9.71% ± 6.29%; individual values: 2.02%, 17.42%, and 9.68%) than in the healthy control (88.10%) and the disease control with chronic active hepatitis B (59.63%), while PPA% was markedly higher in macro-AST patients (mean 90.29% ± 6.28%; individual values: 97.97%, 82.58%, and 90.32%) than in the healthy control (11.90%) and the disease control (40.37%).

**Conclusion:**

Macro-AST should be considered in cases of persistent isolated AST elevation with unremarkable workup. Testing for macro-AST by PEG precipitation should be considered before proceeding to invasive procedures such as liver biopsy. In this study, the PEG precipitation method proved to be a practical adjunctive tool for identifying macro-AST, although its reliability and diagnostic accuracy in a wider population remain to be confirmed.

## 1 Introduction

Aspartate aminotransferase (AST) and alanine aminotransferase (ALT) are widely used clinical biomarkers for hepatocellular injury. These enzymes are released into the bloodstream following hepatic damage and are routinely measured to aid in the diagnosis of liver diseases (Sheth et al., 1998; Abassa et al., 2022; Contreras et al., 2023). Isolated elevation of AST is relatively uncommon in healthy individuals, and one of its benign causes is the presence of macro-AST. In such cases, AST forms high-molecular-weight complexes (approximately 250 kD) through self-aggregation or binding with immunoglobulins, leading to prolonged clearance and persistent elevation of serum AST levels. Although macro-AST is not inherently pathogenic, it can cause artificially increased enzyme activity. Literature reports indicate a prevalence of macro-AST as high as 38.6% in children (Caropreso et al., 2009), although it is considerably less common in adults. Due to limited awareness among clinicians, patients often undergo a diagnostic odyssey, resulting in psychological distress and unnecessary healthcare resource utilization. The simplest diagnostic method is polyethylene glycol (PEG) precipitation, which sedimentates the IgG-AST complexes present in patient serum, leading to a significant decrease in AST activity in the supernatant and thereby enhancing diagnostic certainty.

Currently, most publications on macro-AST are individual case reports, with a lack of systematic summaries of multiple patients. Thus, the clinical characteristics, laboratory patterns, and diagnostic approaches remain poorly defined, particularly among Chinese populations, for which there are scarcely any group reports. In this study, we present three cases of macro-AST in Chinese adults who underwent comprehensive evaluations and were ultimately identified by PEG precipitation. Our primary aim is to elucidate the clinical and laboratory features of macro-AST in this population—for which group data are currently scarce—and to share our practical experience with the PEG precipitation method as an adjunctive diagnostic tool.

## 2 Materials and methods

### 2.1 Case selection and data collection

This was a descriptive observational study presenting a case series of macro-AST in Chinese adults. This study consecutively enrolled patients with persistent isolated elevation of AST who attended the First Department of Integrative Medicine at Beijing Ditan Hospital Affiliated to Capital Medical University between February and August 2025. The inclusion criteria were persistent elevation of AST for more than 6 months, normal ALT, and no clinical evidence of an underlying condition that could explain the elevated AST, as confirmed by comprehensive serological and histological evaluation. All patients underwent systematic evaluation to exclude common causes of elevated transaminases, including chronic viral hepatitis (hepatitis B and C), metabolic dysfunction-associated fatty liver disease, alcoholic liver disease. Imaging examinations included abdominal ultrasound and transient elastography. To further exclude other liver disease, all three patients underwent ultrasound-guided liver biopsy, which showed no specific pathological changes. One patient with chronic active hepatitis B and one healthy volunteer were also included as controls.

Clinical and laboratory data were collected for all subjects, including demographic information (age, sex, medical history, family history), liver function parameters (AST, ALT, alkaline phosphatase [ALP], gamma-glutamyl transferase [GGT], total bilirubin [TBIL], direct bilirubin [DBIL], and total bile acids [TBA]), muscle enzyme profiles (creatine kinase [CK] and lactate dehydrogenase [LDH]), immunological markers (autoantibodies including antinuclear antibody [ANA], anti-mitochondrial antibody [AMA], anti-smooth muscle antibody [SMA], anti-double-stranded DNA antibody [anti-dsDNA], anti-Sjögren’s syndrome-related antigen A antibody [anti-SSA], anti-Sjögren’s syndrome-related antigen B antibody [anti-SSB], anti-Ro-52 antibody [anti-Ro52], anti-mitochondrial M2 antibody [AMA-M2], and anti-nuclear ribonucleoprotein/Smith antibody [anti-nRNP/Sm]; immunoglobulins including IgG, IgG subclasses 1–4, IgA, and IgM; complement components C3 and C4; rheumatoid factor [RF]; and anti-thyroid antibodies including anti-thyroglobulin [anti-Tg] and anti-thyroid peroxidase [anti-TPO]), viral hepatitis markers (hepatitis B surface antigen [HBsAg] and anti-hepatitis C virus antibody [anti-HCV]), and thyroid function tests (thyroid-stimulating hormone [TSH], free triiodothyronine [FT3], and free thyroxine [FT4]). Imaging studies included abdominal ultrasound and transient elastography for liver stiffness measurement; histological evaluation was based on ultrasound-guided liver biopsy pathology.

### 2.2 Biological sample collection and PEG precipitation

Fasting peripheral blood (4 mL) was collected using BD Vacutainer tubes (REF 367812). Samples were transported to the laboratory, balanced, and centrifuged (4 °C, 3000 rpm, 10 min). The supernatant serum was carefully aspirated in a clean bench environment into new centrifuge tubes and placed on ice for subsequent testing.

PEG precipitation was performed following the method described by Maria Lorubbio et al. with minor modifications. Briefly, 100 μL of serum was mixed with 100 μL of 250 g/L PEG 6000 (Solarbio, China) by vortexing. The mixture was centrifuged at 1500 × g for 30 min at 4 °C, and the supernatant was collected as the PEG-AST sample. Another 100 μL of serum was mixed with 100 μL of phosphate-buffered saline (PBS) as the PBS-AST control. AST activity (U/L) was determined using an aspartate aminotransferase assay kit (Nanjing Jiancheng, China) based on the Reitman-Frankel colorimetric method. In this method, AST catalyzes the transamination between α-ketoglutarate and aspartate to generate oxaloacetate, which spontaneously decarboxylates to pyruvate; pyruvate then reacts with 2,4-dinitrophenylhydrazine to form a brown-red complex, measured at 510 nm. Enzyme activity was calculated from a standard curve and expressed in U/L using the conversion factor provided by the manufacturer. AST recovery rate (%) was calculated as (PEG-AST/PBS-AST) × 100 (Patteet et al., 2012). PEG-precipitated activity (PPA%) was calculated as [(PBS-AST − PEG-AST)/PBS-AST] × 100 (Gonzalez Raya et al., 2019). After PEG treatment, the AST recovery rate and PPA% were calculated to evaluate the presence of macro-AST. Data were processed and analyzed using Excel.

### 2.3 Statistical analysis

Clinical and laboratory data were independently entered and verified by two researchers. Data were collated and analyzed using Excel. Measurement data are presented as mean ± standard deviation.

## 3 Results

### 3.1 Baseline characteristics of the patients

Among the three patients, one was male and two were female, aged between 39 and 63 years. The duration from initial detection of AST abnormality to diagnosis ranged from 3 to 17 years. All patients denied a history of rheumatic diseases, as well as relevant family history. The elevated AST was detected incidentally during routine physical examinations, and no related clinical symptoms or positive signs were observed. Baseline characteristics are summarized in **Table 1**, and the complete exclusion work-up, including liver function tests, muscle enzymes, viral markers, thyroid function, and imaging findings, is summarized in **Table 2**. In addition, the detailed immunological profiles are presented in **Table 3**.

**Table 1:** Baseline characteristics of the three patients with macro-AST.

Case No.	Age (years)	Gender	Disease Duration (years)	Past Medical History	Other Positive Signs	Related Treatments
1	39	Female	4	History of laparoscopic surgery for chocolate cyst	/	Traditional Chinese medicine, UDCA
2	51	Male	17	Hyperthyroidism treated with I-131 resulting in hypothyroidism, coronary heart disease	/	Silymarin, Polyene Phosphatidylcholine Capsules
3	63	Female	3	Post-thyroidectomy for thyroid cancer, hypothyroidism	/	Bicyclol, Wuling Capsule, Silymarin

**Table 2:** Complete exclusion work-up for the three patients with macro-AST.

Parameter (reference range)	Case 1	Case 2	Case 3
Liver function			
ALT (U/L) (ref: 7–40)	17.5	29	7.8
AST (U/L) (ref: 13–35)	**98.2**	**83.1**	**121.7**
ALP (U/L) (ref: 35–100)	65.6	85.1	80.5
GGT (U/L) (ref: 7–45)	**6.4**	19.0	16.5
TBIL (μmol/L) (ref: 0–18.8)	14.7	10.1	8.7
DBIL (μmol/L) (ref: 0–6.8)	4.9	4.1	3.0
TBA (μmol/L) (ref: 0–10)	**11.4**	1.3	1.1
Muscle enzymes			
CK (U/L) (ref: 40–200)	74.0	166.3	81.4
LDH (U/L) (ref: 120–250)	190	217.0	181.7
Viral markers			
HBsAg (IU/ml) (ref: <0.05)	0.00	0.00	0.00
Anti-HCV (S/CO) (ref: <1)	0.18	0.06	0.08
Thyroid function			
TSH (uIU/L) (ref: 0.35–4.94)	2.29	2.29	**0.02**
FT3 (pg/mL) (ref: 1.58–3.91)	2.65	2.89	2.55
FT4 (ng/dL) (ref: 0.7–1.48)	1.07	0.99	1.43
Imaging studies			
Abdominal ultrasound	Unremarkable	Unremarkable	Unremarkable
LSM (kPa)	5.6	6.2	5.4
Liver biopsy	Mild non-specific changes (see **Figure 2A**)	Mild non-specific changes (see **Figure 2B**)	Mild non-specific changes (see **Figure 2C**)

ALT, alanine aminotransferase; AST, aspartate aminotransferase; ALP, alkaline phosphatase; GGT, gamma-glutamyl transferase; TBIL, total bilirubin; DBIL, direct bilirubin; TBA, total bile acids; CK, creatine kinase; LDH, lactate dehydrogenase; HBsAg, hepatitis B surface antigen; anti-HCV, hepatitis C virus antibody; TSH, thyroid-stimulating hormone; FT3, free triiodothyronine; FT4, free thyroxine; LSM, liver stiffness measurement. Reference ranges are shown in parentheses. Bold values indicate results outside the reference range. Immunological profiles are detailed separately in **Table 3**. Liver biopsy findings are shown in **Figure 2A** (Case 1), **Figure 2B** (Case 2), and **Figure 2C** (Case 3).

**Table 3:** Immunological profiles of the three patients with macro-AST.

Parameter (reference range)	Case 1	Case 2	Case 3
ANA titer – nuclear speckled (ref: <1:100)	**1:1000**	Negative	Negative
ANA titer – cytoplasmic (ref: <1:100)	**1:100**	Negative	Negative
ANA pattern (overall interpretation)	**Nuclear speckled + cytoplasmic**	Negative	Negative
AMA (ref: Negative)	Negative	Negative	Negative
AMA-M2 (ref: Negative)	**Positive (+++)**	Negative	Negative
SMA (ref: Negative)	Negative	Negative	Negative
anti-dsDNA (ref: Negative)	Negative	Negative	Negative
anti-SSA (ref: Negative)	**Positive (+++)**	Negative	Negative
anti-SSB (ref: Negative)	Negative	Negative	Negative
anti-Ro52 (ref: Negative)	**Positive (+++)**	Negative	Negative
anti-nRNP/Sm (ref: Negative)	**Positive (+)**	Negative	Negative
IgG (g/L) (ref: 7.0–16.0)	**21.00**	10.30	**17.5**
IgG1 (μg/mL) (ref: 3941–10444)	**11117.0**	8515.0	9489.0
IgG2 (μg/mL) (ref: 1661–8064)	5131.0	1815.0	6189.0
IgG3 (μg/mL) (ref: 101–892)	**1035.0**	464.0	826.0
IgG4 (μg/mL) (ref: 36–2090)	337.0	679.0	846.0
IgA (g/L) (ref: 0.7–4.0)	**4.78**	1.16	2.23
IgM (g/L) (ref: 0.4–2.3)	0.89	0.83	1.00
C3 (g/L) (ref: 0.79–1.52)	**0.42**	1.11	1.25
C4 (g/L) (ref: 0.16–0.38)	**0.13**	0.24	**0.49**
RF (IU/ml) (ref: 0–20)	**29**	<20	<20
anti-Tg (IU/ml) (ref: 0–4.11)	**138.72**	1.06	2.31
anti-TPO (IU/ml) (ref: 0–5.61)	1.71	**19.23**	1.89

ANA, antinuclear antibody (detected by indirect immunofluorescence on HEp-2 cells at 1:100 screening dilution; positivity defined as ≥1:100); AMA, anti-mitochondrial antibody; SMA, anti-smooth muscle antibody; anti-dsDNA, anti-double-stranded DNA antibody; anti-SSA, anti-Sjögren’s syndrome-related antigen A antibody; anti-SSB, anti-Sjögren’s syndrome-related antigen B antibody; anti-Ro52, anti-Ro-52 antibody; AMA-M2, anti-mitochondrial M2 antibody; anti-nRNP/Sm, anti-nuclear ribonucleoprotein/Smith antibody; RF, rheumatoid factor; anti-Tg, anti-thyroglobulin antibody; anti-TPO, anti-thyroid peroxidase antibody. Reference ranges are shown in parentheses. Bold values indicate results outside the reference range. (Positive results for anti-SSA, anti-Ro52, AMA-M2, and anti-nRNP/ Sm are reported with semi-quantitative intensities in parentheses).

### 3.2 Immunological findings

Comprehensive autoantibody screening and serum immunoglobulin measurement were performed in all patients, with results summarized in **Table 3**. Case 1 showed the most prominent autoimmune features, with positive ANA displaying two distinct immunofluorescence patterns on HEp-2 cells—nuclear speckled at 1:1000 and cytoplasmic at 1:100—along with multiple positive autoantibodies (anti-SSA [+++], anti-Ro52 [+++], AMA-M2 [+++], and anti-nRNP/Sm [+]), elevated total IgG (21.0 g/L) with elevated IgG1 and IgG3 subclasses, elevated IgA (4.78 g/L), decreased C3 (0.42 g/L) and C4 (0.13 g/L), positive RF (29 IU/mL), and markedly elevated anti-Tg (138.72 IU/mL). Case 2 was negative for all autoantibodies tested except for anti-TPO (19.23 IU/mL), with normal immunoglobulin levels and complements. Case 3 was negative for all autoantibodies but showed mildly elevated total IgG (17.5 g/L) with normal IgG subclasses, and elevated C4 (0.49 g/L). These findings indicate that all three macro-AST patients exhibited different degrees of immune disorders with obvious heterogeneity in immune phenotypes.

### 3.3 Dynamic changes in AST levels and liver histopathology

All patients had received various liver-protective medications due to elevated AST (**Table 1**), yet AST levels remained persistently abnormal. At the time of presentation to our hospital, AST levels ranged from 83.1 to 121.7 U/L. As shown in **Figure 1**, historical AST levels in all three patients consistently exceeded the upper limit of normal, while ALT levels remained normal throughout. Even with additional liver-protective agents (e.g., Patient 2 received two agents), AST levels did not decrease and sometimes increased further (e.g., Patient2: 96 U/L), suggesting that the AST elevation was not due to typical hepatocellular injury. Pathological assessment of the liver biopsies revealed only mild, non-specific changes (mild bile duct injury, lymphocytic infiltration, steatosis, and spotty necrosis), none of which adequately explained the persistent isolated AST elevation (**Figure 2**).

**Figure 1:**
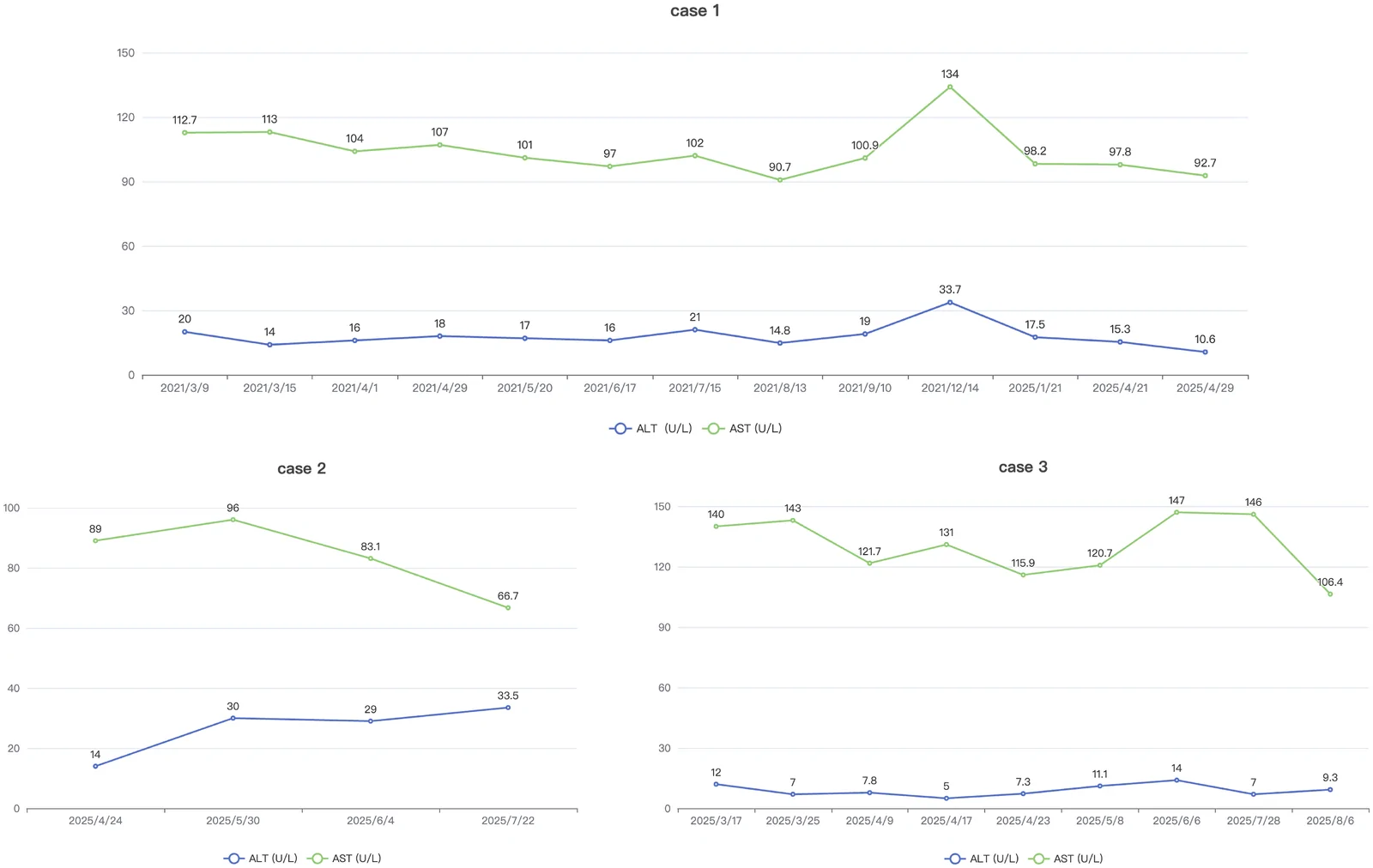
Dynamics of serum ALT and AST levels in the three patients with macro-AST.

**Figure 2:**
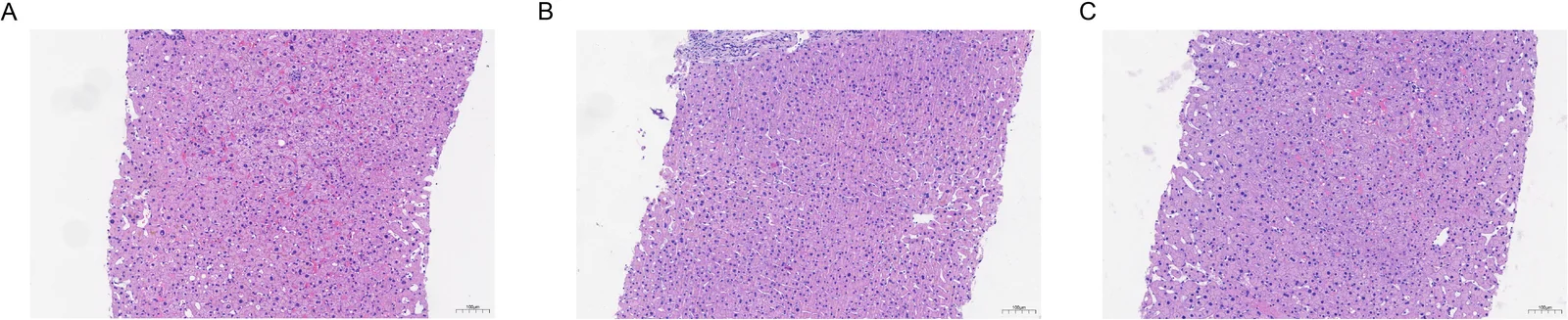
Liver biopsy findings from three patients with macro-AST (hematoxylin and eosin staining; original magnification 100×; scale bar = 100 μm). **(A)** Case 1: Mild interlobular bile duct injury in the portal area with lymphocytic infiltration, along with mixed macrovesicular and microvesicular steatosis in hepatocytes. **(B)** Case 2: Mild lymphocytic infiltration in sinusoids and portal areas, mild interlobular bile duct injury, and reactive bile duct hyperplasia at the peripheral region. **(C)** Case 3: Occasional mixed macrovesicular and microvesicular steatosis (<5%) in zone III of the hepatic lobules, scattered spotty necrosis, and mild lymphocytic infiltration with occasional plasma cells in the portal area, without interface hepatitis. Despite these mild histological abnormalities, none of the findings adequately explained the persistent isolated AST elevation in these patients.

### 3.4 Detection of macro-AST by PEG precipitation

Given the unexplained isolated AST elevation and considering macro-AST as benign cause, we employed the PEG precipitation method described by Maria Lorubbio et al. to detect the presence of macro-AST in serum. A standard curve was generated based on the kit instructions, yielding the equation: y = 19458x³ - 2983.8x² + 634.54x - 0.6987 (R² = 0.9989). Serum-AST, PEG-AST, and PBS-AST were measured using the Nanjing Jiancheng kit (Reitman-Frankel method), and AST recovery rate and PPA% were calculated. It should be noted that these laboratory AST values were obtained using a different methodology from the clinical AST measurements and are therefore not directly comparable in absolute terms (see **Table 4** footnote).

**Table 4:** Laboratory-AST parameters in the three patients, disease control, and healthy control subjects.

Parameter		Case		Case control	Healthy control
	Case1	Case2	Case3		
Serum-AST (U/L)	19.05	15.43	15.18	35.36	10.11
PEG-AST (U/L)	0.44	3.21	1.43	18.18	7.92
PBS-AST (U/L)	21.72	18.43	14.77	30.49	8.99
AST recovery rate (%)	2.02	17.42	9.68	59.62	88.10
PPA%	97.97	82.58	90.32	40.37	11.90

Laboratory AST values were measured using the Reitman-Frankel colorimetric method (Nanjing Jiancheng kit) and are expressed in U/L based on the manufacturer’s conversion factor. AST recovery rate (%) = (PEG-AST/PBS-AST) × 100; PPA % = [(PBS-AST − PEG-AST)/PBS-AST] × 100. PPA, polyethylene glycol-precipitable activity; PEG, polyethylene glycol; PBS, phosphate-buffered saline.

As shown in **Table 4**, Case 1 showed an AST recovery rate of 2.02% and PPA% of 97.97%; Case 2, 17.42% and 82.58%; Case 3, 9.68% and 90.32%. All three patients exhibited PPA% values far exceeding the various cutoffs proposed in the literature (e.g., >73% by González Raya et al (Gonzalez Raya et al., 2019); 61% by Bürki et al (Burki et al., 2023); and >71% in confirmed macro-AST cases by Davidson and Watson (2003)), supporting the presence of macro-AST. The disease control (chronic active hepatitis B) had an AST recovery rate of 59.63% and PPA% of 40.37%, while the healthy control showed 88.10% and 11.90%, respectively. These results illustrate the practical application of the PEG precipitation assay in identifying macro-AST in this case series.

## 4 Discussion

Macro-AST is a relatively uncommon clinical phenomenon with an unclear pathogenesis, and only a limited number of cases have been reported in the literature. Its long-term impact on disease progression and other pathological consequences remains unknown, often leading to its oversight in clinical practice. Macro-AST belongs to a group of substances known as macro enzymes—normal serum enzymes that form high-molecular-weight complexes. Due to their size, they cannot be cleared renally, leading to their persistence in the blood, which interferes with routine measurements and causes diagnostic confusion (Pascarella et al., 2007).

Like other macro enzymes, macro-AST can be classified into two types. In Type I, the mechanism appears to be related to autoimmunity, where the Fab portion of immunoglobulins (IgG, IgM, or IgA) targets the serum enzyme as an antigen in a context of molecular mimicry. In Type II, the macro enzyme forms transiently through self-aggregation of the enzyme or association with foreign chemicals, such as drugs or the apoprotein component of lipoprotein carriers (Lee et al., 2011; Luk et al., 2019). The prevalence of most macro enzymes is low, ranging from 0.5% to 2.5% (Sturk and Sanders, 1990). Their presence has been described in various diseases, including chronic hepatitis C and autoimmune disorders (Zhan et al., 2019; Schimming et al., 2021; Simac et al., 2021).

Diagnosis of macro-AST is typically one of exclusion. Clinicians should be aware of this condition and maintain a high index of suspicion. A comprehensive patient history and routine laboratory tests are essential to rule out other related or coexisting conditions that could explain the elevated AST levels. Confirmatory diagnostic tests include protein electrophoresis, PEG precipitation, gel filtration chromatography, or pyridoxal-5-phosphate activation assays (de Jonge and Houwing, 1996; Davidson and Watson, 2003; Wener et al., 2012).

In this study, we employed the PEG precipitation method due to its simplicity, low cost, and effectiveness in separating macromolecular complexes such as AST-immunoglobulin complexes. It should be noted that no universally standardized diagnostic criteria for macro-AST by PEG precipitation have been established to date. Various studies have employed different thresholds: González Raya et al. proposed PPA > 73% as the diagnostic cutoff, while others have defined positivity based on AST recovery rate ≤ 40% (Gonzalez Raya et al., 2019; Lorubbio et al., 2020). A recent large-scale study established enzyme-specific 99th percentile cutoffs for %PPA, with AST at 61%, and suggested that %PPA ≥ 80% makes the presence of a macroform very likely for all enzymes (Burki et al., 2023). This heterogeneity underscores the need for further standardization. In the present study, all three patients exhibited PPA values (97.97%, 82.58%, and 90.32%) substantially exceeding all proposed cutoffs, providing robust evidence for the diagnosis. The diagnosis of macro-AST was confirmed by a markedly decreased AST recovery rate along with an elevated PPA% in the patients compared to both healthy controls and disease controls. Our three patients exhibited markedly low AST recovery (9.71 ± 6.29%) and high PPA% (90.29 ± 6.28%), in clear contrast to the disease controls (59.63% and 40.37%, respectively) and healthy controls (88.10% and 11.90%, respectively), further validating the presence of macro-AST. It is also worth noting that the AST activities measured in the laboratory for PEG precipitation experiments (15.18-19.05 U/L) were substantially lower than the clinical AST values (83.1-121.7 U/L) from the same patients. This discrepancy reflects the use of different analytical methods—the Reitman-Frankel colorimetric method for the laboratory experiments versus the IFCC ultraviolet kinetic method for routine clinical testing—rather than any true change in the patients’ AST levels. As highlighted in **Table 3**, these values are derived from different calibration systems and should not be directly compared.

Macro-AST adds both financial and psychological burden to patients. For instance, Case 1, after detecting isolated AST elevation in 2021, underwent repeated outpatient visits and treatments due to anxiety, incurring substantial costs. Following a confirmed diagnosis of macro-AST, the patient’s psychological distress alleviated significantly, and unnecessary medical visits ceased. Currently, the patient only requires regular follow-up with liver enzyme monitoring, particularly given the concurrent presence of ANA antibodies. These three confirmed cases have enhanced physicians’ awareness of macro-AST as a cause of isolated AST elevation at our hospital. We emphasize that in patients with persistent isolated AST elevation, after common hepatic and extrahepatic causes have been excluded, testing for macro-AST by PEG precipitation should be considered before proceeding to invasive procedures such as liver biopsy.

We acknowledge several limitations in this study. First, as a case series with only three macro-AST patients, our sample size is small, which limits the generalizability of our findings. Second, all cases were from a single center, and selection bias may exist. Third, the inclusion of only one disease control (chronic active hepatitis B) and one healthy control was intended for illustrative comparison rather than for formal diagnostic validation; therefore, the diagnostic performance of the PEG precipitation method—such as sensitivity, specificity, or optimal cut-off values—cannot be established from this study. Larger, multi-center studies with independent confirmatory methods are warranted to further validate this approach. Fourth, we did not perform additional independent confirmatory tests—such as gel filtration chromatography, protein electrophoresis, or immunofixation—to directly visualize or characterize the AST-immunoglobulin complexes. While PEG precipitation is a widely used and practical screening tool for macroenzyme detection, and the markedly reduced AST recovery and elevated PPA values in our patients were highly suggestive of macro-AST, the absence of a second confirmatory technique remains a methodological limitation. Fifth, the laboratory AST measurements for the PEG experiments (performed using the Reitman-Frankel colorimetric method) and the clinical AST measurements (performed using the IFCC ultraviolet kinetic method) were obtained using different analytical systems, with different reaction temperatures (25 °C vs. 37 °C) and unit definitions. Therefore, the absolute AST values from the two methods are not directly comparable, although this does not affect the calculation of AST recovery rates or PPA%, as each was derived using the same method for pre- and post-PEG comparisons.

## 5 Conclusion

Macro-AST is a rare but important benign cause of persistent isolated AST elevation. In patients with persistent isolated AST elevation, after common hepatic and extrahepatic causes have been excluded, testing for macro-AST by PEG precipitation should be considered before proceeding to liver biopsy. The PEG precipitation method is a simple, non-invasive, and cost-effective adjunctive tool that can aid in the identification of macro-AST and help avoid unnecessary invasive procedures.

## Data Availability

The raw data supporting the conclusions of this article will be made available by the authors, without undue reservation.
