# Correction: Solving the mystery of persistent isolated aspartate aminotransferase elevation: Macro-AST as a decisive diagnostic consideration

**DOI:** 10.3389/fcimb.2026.2022545

**Published:** 2026-10-15

**Authors:** Lina Sun, Yao Liu, Xue Yang, Fangyuan Gao, Lei Sun, Yu Lu, Yanqiu Li, Ying Feng, Xianbo Wang

**Affiliations:** 1 Center of Integrative Medicine, Beijing Ditan Hospital, Capital Medical University, Beijing, China; 2 National Center for Infectious Diseases, Beijing Ditan Hospital, Capital Medical University, Beijing, China; 3 Department of Pathology, Beijing Ditan Hospital, Capital Medical University, Beijing, China

**Keywords:** AST recovery rate, immune dysregulation, macro-AST, PEG precipitation, PPA%

In the published article,

Affiliation 2 ”National Center for Infectious Diseases, Beijing Ditan Hospital, Capital Medical University, Beijing, China” was omitted from the paper. This affiliation has now been added for author “Lina Sun”.

Affiliation 3 “Department of Pathology, Beijing Ditan Hospital, Capital Medical University, Beijing, China” was omitted from the paper. This affiliation has now been added for author “Lei Sun”.

Affiliation 3 “Department of Pathology, Beijing Ditan Hospital, Capital Medical University, Beijing, China” was erroneously included in the paper. This affiliation has now been removed for author “Lina Sun”.

Affiliation 2 ”National Center for Infectious Diseases, Beijing Ditan Hospital, Capital Medical University, Beijing, China” was erroneously included in the paper. This affiliation has now been removed for author “Lei Sun”.

The original version of this article has been updated.

